# Vascular and haemodynamic issues of brain ageing

**DOI:** 10.1007/s00424-020-02508-9

**Published:** 2021-01-13

**Authors:** Lucy Beishon, Rebecca H. Clough, Meeriam Kadicheeni, Tamara Chithiramohan, Ronney B. Panerai, Victoria J. Haunton, Jatinder S. Minhas, Thompson G. Robinson

**Affiliations:** 1 Department of Cardiovascular Sciences, University of Leicester, Robert Kilpatrick Clinical Sciences Building, Leicester, LE2 7LX UK; 2 NIHR Leicester Biomedical Research Centre, British Heart Foundation Cardiovascular Research Centre, Glenfield Hospital, Leicester, UK

**Keywords:** Dynamic cerebral autoregulation, Neurovascular coupling, Transcranial Doppler ultrasonography, Ageing

## Abstract

The population is ageing worldwide, thus increasing the burden of common age-related disorders to the individual, society and economy. Cerebrovascular diseases (stroke, dementia) contribute a significant proportion of this burden and are associated with high morbidity and mortality. Thus, understanding and promoting healthy vascular brain ageing are becoming an increasing priority for healthcare systems. In this review, we consider the effects of normal ageing on two major physiological processes responsible for vascular brain function: Cerebral autoregulation (CA) and neurovascular coupling (NVC). CA is the process by which the brain regulates cerebral blood flow (CBF) and protects against falls and surges in cerebral perfusion pressure, which risk hypoxic brain injury and pressure damage, respectively. In contrast, NVC is the process by which CBF is matched to cerebral metabolic activity, ensuring adequate local oxygenation and nutrient delivery for increased neuronal activity. Healthy ageing is associated with a number of key physiological adaptations in these processes to mitigate age-related functional and structural declines. Through multiple different paradigms assessing CA in healthy younger and older humans, generating conflicting findings, carbon dioxide studies in CA have provided the greatest understanding of intrinsic vascular anatomical factors that may mediate healthy ageing responses. In NVC, studies have found mixed results, with reduced, equivalent and increased activation of vascular responses to cognitive stimulation. In summary, vascular and haemodynamic changes occur in response to ageing and are important in distinguishing “normal” ageing from disease states and may help to develop effective therapeutic strategies to promote healthy brain ageing.

## Introduction

The population is ageing, and by 2050, one in six people will be aged over 65 worldwide [75]. Cerebrovascular disease is a prominent cause of morbidity and mortality, and the prevalence of the cerebrovascular disease is increasing with the ageing population [123]. Indeed, 15 million people worldwide are affected by stroke, 60% of whom are aged over 70 [123] and 46.8 million affected by dementia [98]. Many research studies have focussed on the vascular and haemodynamic changes involved in cerebrovascular disease. However, it is important to first understand the haemodynamic changes associated with normal ageing, in order to delineate these from pathological changes that may occur in disease states.

There are multiple techniques that can be employed to assess the vasculature and haemodynamics of the brain. Transcranial Doppler ultrasonography (TCD) allows for the assessment of cerebral haemodynamics in a non-invasive manner, by continuous monitoring of beat-to-beat cerebral blood flow (CBF) velocity as an approximation of CBF [85]. Near-infrared spectroscopy (NIRS) exploits the differences in infrared spectra absorption between oxygenated (oxyHb) and deoxygenated haemoglobin (dexoyHb) to measure relative changes in their concentration [23]. NIRS can therefore be used to measure tissue oxygenation and as an indirect measure of CBF [23]. Furthermore, functional magnetic resonance imaging (fMRI) and positron emission tomography (PET) can be used indirectly to measure neural activation [3].

An important factor of vascular ageing is arterial stiffness, which is known to increase with age and can be a predictor of end-organ damage [52]. Arterial stiffness is most commonly assessed using pulse wave velocity (PWV), directly at the level of the aorta, or between the carotid-femoral or brachial-ankle arterial sites [131]. Longitudinal epidemiological studies have shown arterial stiffness can progress at the average rate of 0.2 to 0.7 m/s for every 5 years of life [52]. Studies of note include the Whitehall II, which measured carotid-femoral PWV in 3789 men and 1383 women free from cardiovascular disease, every 4 years [2]. Results showed an increase in arterial stiffness, with an exponential increase in the progression with age [2]. Similar results were found in the SaridNIA study [107]. Increasing arterial stiffness may be due to a disturbance in the regulatory pathways involved in sustaining the arterial extracellular matrix [107]. For example, upregulation of the renin-angiotensin system results in an increase in activation of pro-inflammatory pathways and thus increased destruction of the vessel wall [107]. Furthermore, fragmentation of elastin fibres, alongside increased collagen production, results in reduced elasticity of the vessel wall and ultimately arterial stiffness [107]. Chronic hypoperfusion as a result of narrowed, less responsive vessels leads to microvascular ischaemia, which can result in brain atrophy and tissue damage over time [43, 45]. The changes with age in PWV and central pulse pressure result in a reduced ability of the larger cerebral vessels to dampen pulsatile energy, which is transmitted directly to the smaller brain vasculature [52]. This combined with elevated vascular risk factors (e.g. hypertension, raised cholesterol), results in progressive damage to smaller vessels [52]. Small vessel disease can lead to chronic conditions such as vascular dementia, as well as acute incidents such as lacunar infarctions [52].

In addition to arterial stiffness, endothelial dysfunction is thought to be a significant contributor to cerebrovascular ageing and results from a reduction in nitric oxide (NO) availability, oxidative stress and chronic inflammation [108]. Endothelial cells are important in the regulation of vascular tone, and thus CBF, through the release of NO (a potent vasodilator) [50]. Furthermore, endothelial dysfunction may contribute to age-related disorders, such as dementia [50, 130], and can be improved with healthy lifestyle interventions [108].

**Fig. 1:**
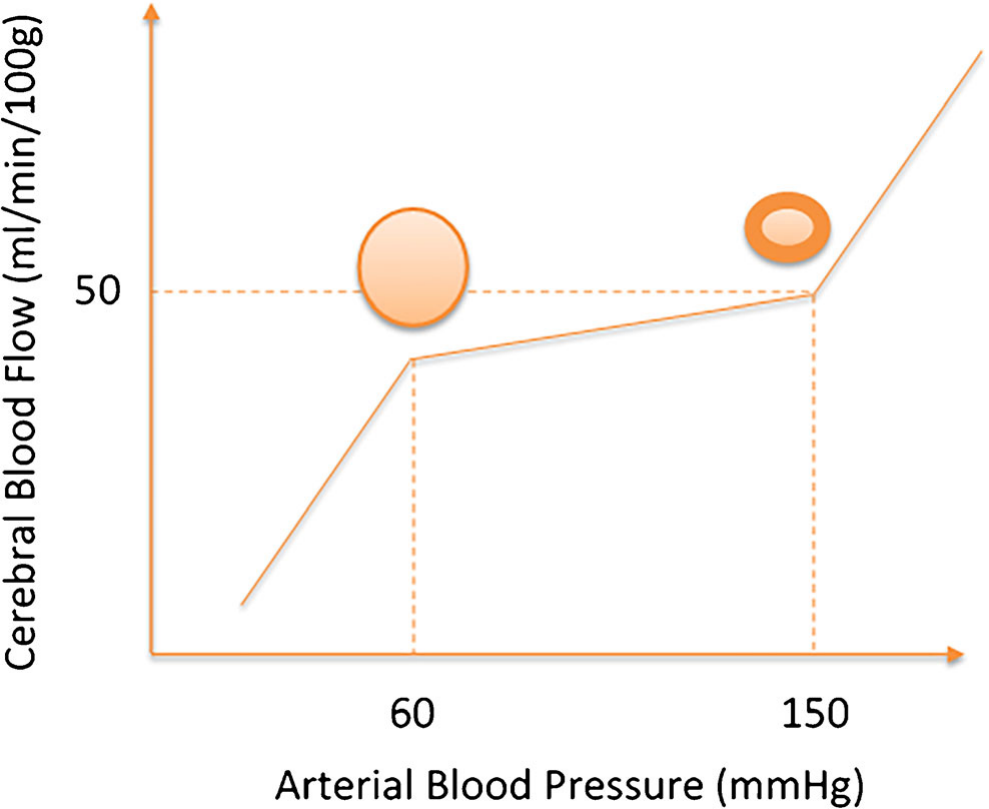
Schematic representation of the concept of static CA as originally proposed by Lassen [53], showing the lower and upper mean blood pressure limits of CA. Arterioles are maximally vasodilated at and below the lower limit and maximally constricted at the upper limit, as represented by the cross sections in the diagram. Recent studies have suggested that the regions where static CA is active show a slope greater than zero and that this could be different for increases or reductions in mean blood pressure [77]

The brain relies on CBF to sustain neuronal metabolism due to its high level of metabolic activity and limited capacity for storage [118]. The cerebrovasculature has a low inherent ability to cope with the high volume blood supply, making the brain vulnerable to changes in CBF [118]. Therefore, cerebral autoregulation (CA) is important in sustaining a relatively constant CBF despite changes in arterial blood pressure (ABP) [53]. Lassen described a segmental autoregulatory curve to conceptualise CA (Fig. 1) [53]. The curve describes a lower limit, below which there is a risk of hypoperfusion, a plateau phase in which changes in perfusion pressure are unlikely to cause damage, and an upper limit above which risks structural damage to the brain [53]. The Rotterdam study followed 1730 participants aged over 50 years without cardiovascular disease and found CBF declined with normal ageing and to a higher degree in men than women [103]. This reduction in CBF can be associated with a decrease in cerebral metabolic rate, affecting neuronal activities in the brain [55].

Neural processing is highly resource intensive, requiring ~ 20% of the body’s resting energy demands, making the brain one of the most metabolically active organs in the body [57, 117]. Under normal physiological conditions, CBF is tightly coupled to neuronal activity through the process of NVC [32], ensuring rising metabolic needs are met during times of increased neuronal activity [23, 57, 117]. NVC must occur in a coordinated fashion, ensuring that CBF is increased in a region-specific manner, diverting flow to areas involved with specific neuronal processes and functions (functional hyperaemia) [32, 117]. This coordination is achieved through the neurovascular unit, which is formed structurally and functionally by neuronal, vascular endothelial, smooth muscle and supporting cells (astrocytes, glia) [32, 117, 137].

**Fig. 2:**
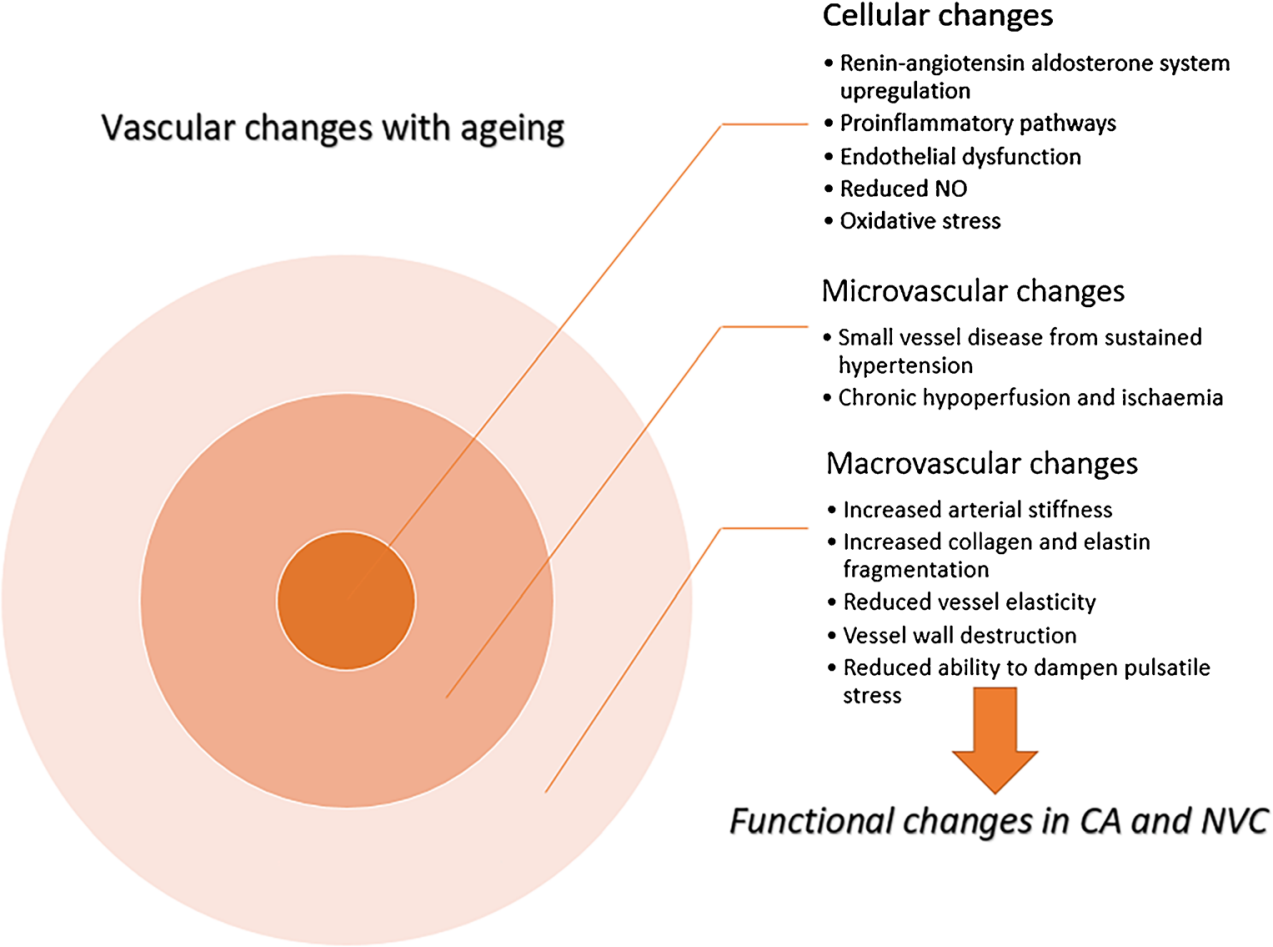
The vascular changes and mechanisms seen with ageing that may lead to functional alterations in cerebral autoregulation (CA) and neurovascular coupling (NVC). NO, nitric oxide; CA, cerebral autoregulation; NVC, neurovascular coupling

The framework for this review is based on the theory that the structural and cellular changes outlined above will result in functional changes in vascular physiology. In particular, CA and NVC are two functional vascular processes critical for the maintenance of adequate brain function. Figure 2 conceptualises the framework and basis of age-related vascular changes leading to impairments in vascular function. This review will now focus on these two key processes in further detail below.

## Neurovascular coupling

**Fig. 3:**
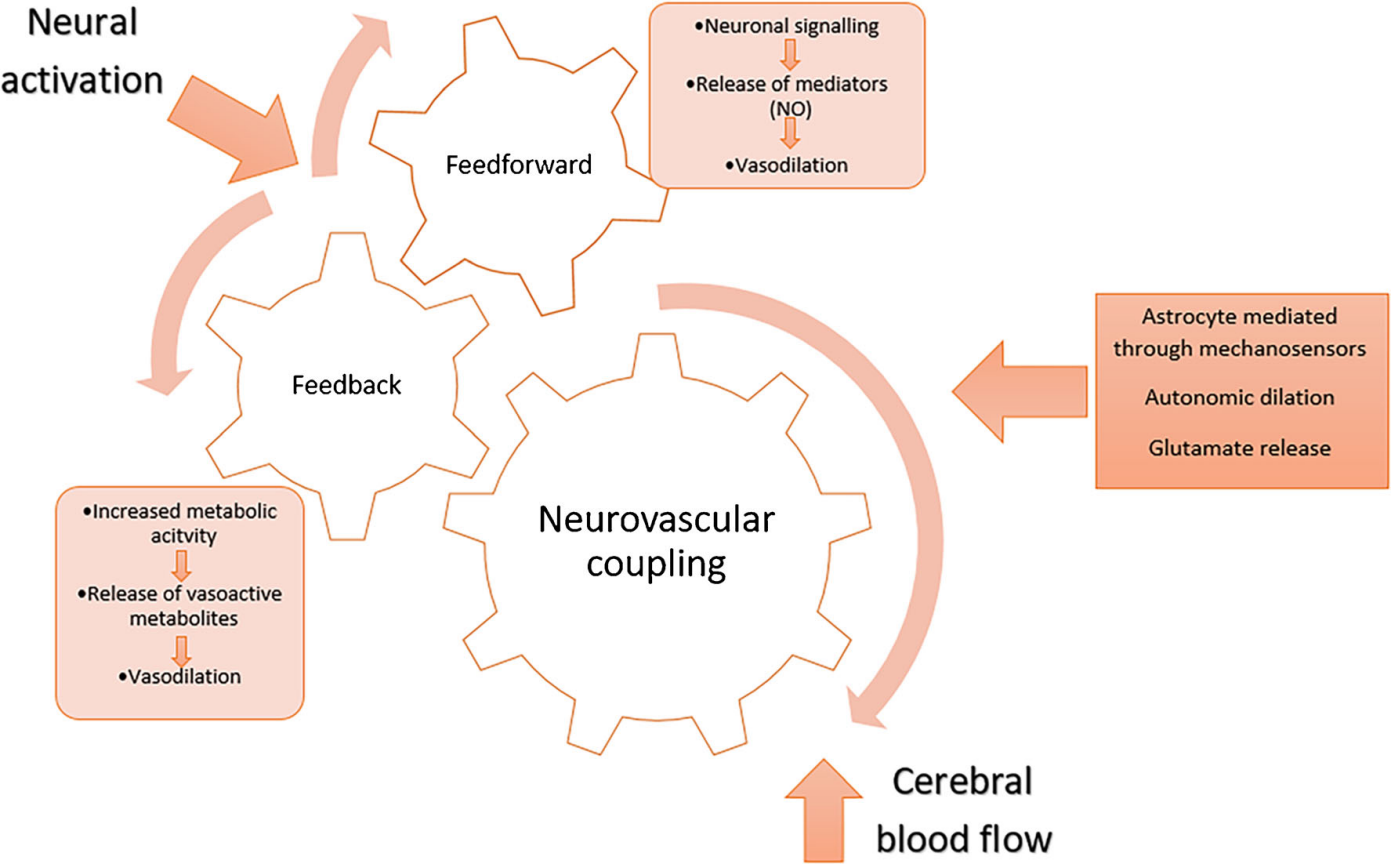
Feedforward and feedback mechanisms that may result in NVC-mediated increases in cerebral blood flow following neural activation. NO, nitric oxide

NVC is mediated by three main mechanisms [97, 113]. Firstly, metabolic activity generates vasoactive metabolites (e.g. CO_2_, lactate) which provide “feedback” resulting in vessel dilation and thus increased flow [38, 40, 97, 113, 137]. However, a “feedforward” model has also been proposed, whereby neuronal signalling drives NVC through the release of mediators resulting from synaptic activity (e.g. K+, NO) and prostanoids [40]. Recently, astrocytes have been found to be sensitive to local changes in oxygen, CO_2_, lactate, pH and neurotransmitter release as a result of metabolic activity and may act as an intermediary between tissue pressure and neural activity through mechanosensors [38, 40]. Shear wall stress occurs when flow increases through a vessel, applying a physical stress to the vessel wall, resulting in the release of vasoactive mediators, such as nitric oxide, resulting in vasodilation [113]. Finally, the autonomic nervous system is able to constrict and dilate arteriolar smooth muscle by altering the level of sympathetic and parasympathetic tone, respectively [97, 113, 137]. Glutamate stimulates neurones and astrocytes to release chemical mediators (i.e. NO, prostaglandin, potassium), which act on vascular smooth muscle to cause vasodilation [39, 40, 67]. Figure 3 shows the integration of feedforward and feedback mechanisms proposed in NVC processes resulting in increased CBF.

A recent development in our understanding of NVC has been the identification of pericytes, which are located in close proximity to capillaries in the brain [35]. Pericytes are contractile cells that may have the ability to regulate capillary vessel diameter and thus influence flow at the capillary bed [35]. However, in vivo studies are less clear about the role of pericytes in modulating NVC, and their role may be more to re-distribute flow at a local level [39].

A number of age-related changes occur in NVC processes, but the shift from normal age-related change to that associated with age-related diseases (e.g. Alzheimer’s dementia, Parkinson’s disease) is less well understood. Thus, understanding the changes that occur under normal ageing, and how these may subsequently evolve into age-related disorders, is imperative to develop effective treatments for the maintenance and promotion of healthy brain ageing.

## Measurement of NVC in humans

NVC can be measured indirectly using a number of non-invasive neuroimaging techniques [39], where individuals are stimulated using visual, auditory, motor, sensory or cognitive paradigms to elicit a rise in metabolic activity [33, 115]. Traditionally, functional magnetic resonance imaging (MRI) used blood oxygen level-dependent (BOLD) signal to measure changes in the concentration of deoxygenated haemoglobin (dexoyHb) in response to cognitive stimulation, as a proxy for metabolic activity [11, 33, 39]. Where participants are cognitively stimulated, deoxyHb levels rise initially as the limited oxygen stores are consumed [25, 33]. This is followed by a fall in deoxyHb concentration as NVC processes work to deliver oxygen, usually in excess of the amount which has been utilised [33]. However, BOLD fMRI is limited to measurement of oxygen concentration and cannot provide any information on the haemodynamics of the NVC response [11, 33]. Arterial spin labelling (ASL) fMRI, which magnetically labels water as an endogenous tracer, rather than measuring changes in blood oxygenation, does allow for quantification of flow measurements [11, 33, 37]. However, widespread adoption of ASL techniques has been hampered by low signal to noise ratio, and lack of standardisation in methods and techniques between centres, although recent efforts have been made to overcome these limitations [33, 37]. BOLD and ASL fMRI have an excellent spatial resolution (1–2 mm) but lack the temporal resolution (1–2 s vs. 5 ms) of alternatives, such as TCD [39]. Furthermore, BOLD fMRI is susceptible to misinterpretation in older adults given a number of age-related effects on the signal including increased signal variability [46], lower resting CBF [23], reduced grey matter volume and altered vascular structure and function [46]. Importantly, baseline measurements prior to task activation are important in studies of ageing. Given that older adults are more likely to have lower resting flow measurements, task-activated responses can appear greater compared to younger adults as a result of lower resting values, if baseline differences are not accounted for [25]. ASL measurements can be useful in this setting to clarify the difference in resting and activated flow states that can only be measured by change from baseline using other techniques (e.g. BOLD-MRI) [25]. Correction for partial volume effects is not undertaken by all studies and may also account for differences in hyper- and hypoactivation [46].

NIRS measures the relative fluctuations of deoxyHb and oxygenated haemoglobin (oxyHb) to determine changes in CBF mediated via NVC processes [23]. Where participants are cognitively stimulated, the relative concentration of oxyHb will rise as CBF increases and the change in oxyHb/deoxyHb can be measured through their different absorption wavelengths in the infrared spectra (~ 700–900 nm) [23, 49]. Whilst NIRS can measure the change in oxygenation, it cannot directly measure the change in CBF [23]. Furthermore, NIRS has a limited penetration depth and thus can only measure superficial and not deeper cortical changes in response to stimulation [49].

TCD uses ultrasound to measure changes in CBF velocity (CBFv) following cognitive stimulation [6, 115]. CBFv is measured as a proxy for CBF on the assumption that the vessel diameter remains relatively constant under small fluctuations in CO_2_ and blood pressure [15, 85]. TCD can be used to measure NVC in the middle, anterior and posterior cerebral arteries (MCA, ACA and PCA, respectively) [85]. Thus, as TCD measure changes in larger cerebral vessels, it has excellent temporal, but limited spatial, resolution [85]. Similar to ASL, TCD is advantageous in its ability to provide a measure of haemodynamic changes, rather than relying on indirect measures of changes in oxy/deoxyHb. In the context of ageing, NIRS and TCD are advantageous in their portability, relative lower cost and use in patients with pacemakers and metal implants [23, 49].

Dynamic retinal vessel analysis (DVA) is a relatively newer technique which examines the change in retinal arteriolar vessel diameter following light exposure using a retinal camera [57]. Retinal vessels are both structurally and functionally similar to their cerebral counterparts, but non-invasive imaging of the retinal vessels can be achieved relatively easily and cheaply when compared to cerebral vessel imaging [57]. NVC processes in the retinal vessels can be measured following light stimulation and invoke similar processes to those occurring in cerebral tissue (glial cell activation, vasodilation) [57]. Furthermore, retinal vessels are susceptible to similar pathological processes as cerebral vessels and abnormalities in the retinal vasculature are predictive of cerebrovascular pathology (stroke, vascular dementia) [24, 57].

## NVC in ageing

**Fig. 4:**
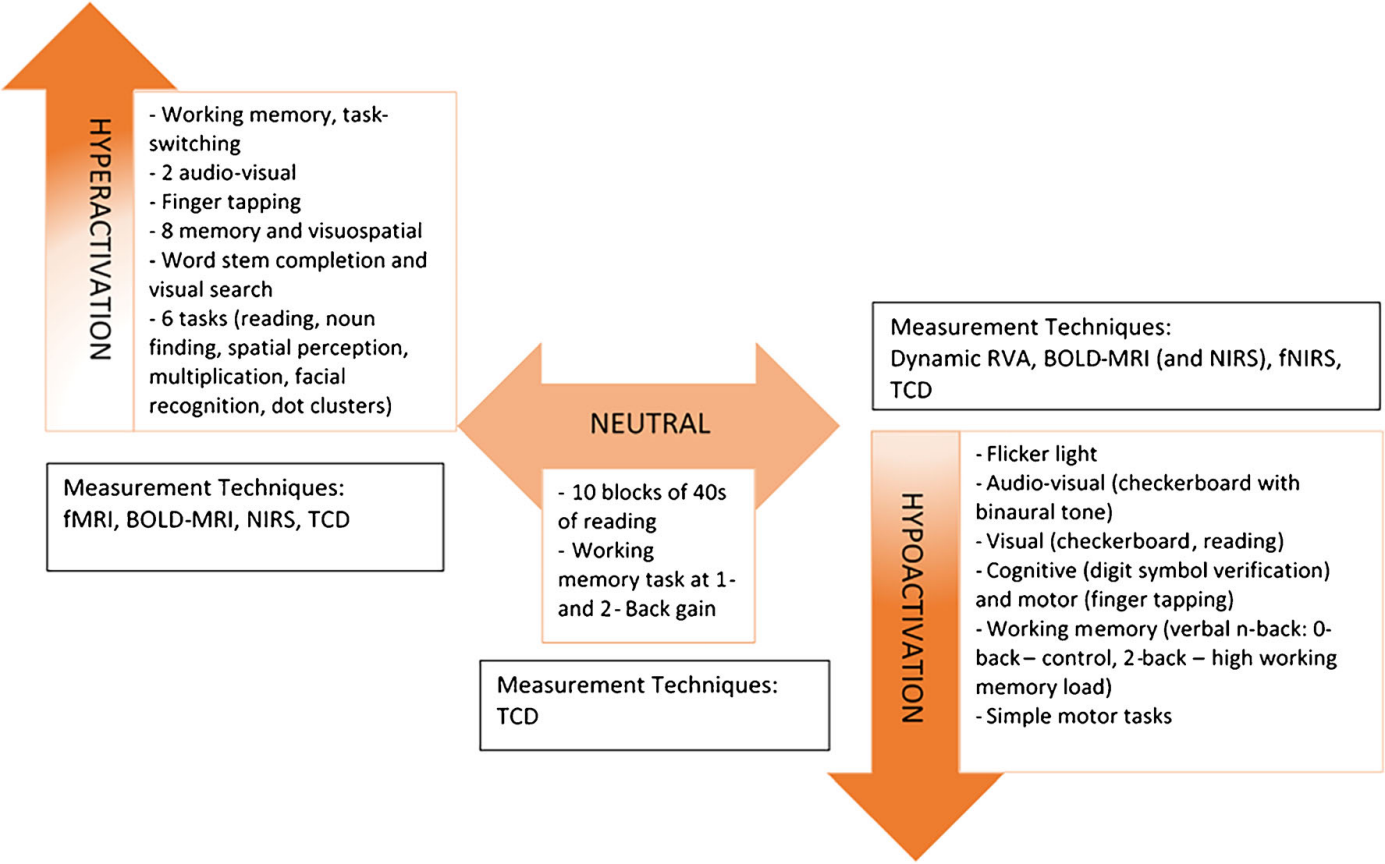
Tasks showing changes in NVC responses in ageing using various measurement techniques. fMRI, functional magnetic resonance imaging; BOLD-MRI, blood oxygenation level-dependant magnetic resonance imaging; NIRS, near-infrared spectroscopy; TCD, transcranial Doppler ultrasonography; fNIRS, functional near-infrared spectroscopy; RVA, retinal vessel analysis

Studies have found conflicting effects of ageing on NVC processes. We consider these under three major headings: hyperactivation, neutral activation and hypoactivation. Table 1 and Fig. 4 summarise the characteristics, techniques and major findings of the studies in this review.

**Table 1:** Summary of the characteristics and main findings of the NVC studies included in this review.

Study ID	Imaging modality	Number of participants	Age of participants (years)	Task activation method	Main findings
Hyperactivation					
Jamadar [42]	fMRI	17 older, 19 younger	Mean ages: 71 and 22	Working memory, task-switching (four difficulty levels)	Increased activation in the right caudate nucleus older compared to younger adults. At the lower difficulty level, younger adults had increased activation, no differences between groups at an intermediate difficulty and older adults showed increased activity at high difficulty level compared to younger adults.
Grinband [34]	BOLD-MRI	34 older, 55 younger	Mean ages: 65 and 25	2 audio-visual tasks	No significant effect of ageing on the time course of the haemodynamic response or the slope of the BOLD vs. stimulus duration relationship
Csipo [23]	NIRS	13 older, 11 younger	Mean ages: 76 and 32	Finger tapping	Increased haemodynamic response in the contralateral motor and prefrontal cortices in older adults. Delayed and reduced deoxyHb signal, loss of early oxyHb signal
Beishon [7]	TCD (MCA)	25 older, 29 younger	Median ages: 64 and 22	8 memory and visuospatial tasks from the Addenbrooke’s cognitive examination-III	Increased peak percentage change in CBFv in older adults in memory (2.17 vs. 8.38%) and visuospatial (5.87 vs. 11.89%, 6.30 vs. 11.30%) tasks
Sorond [112]	TCD (ACA and PCA)	15 older, 14 younger	Mean ages: 74 and 30	Word-stem completion and visual search tasks	Increased CBFv responses in older compared to younger adults. Loss of regional activation in older group for word-stem completion task
Droste [26]	TCD (MCA)	33 older, 37 younger	Older > 30 years old, younger < 30 years old	6 tasks encompassing reading, finding nouns, spatial perception, multiplication, facial recognition and assigning dot clusters to a distance scale	Increased CBFv responses to task activation in older adults, but resting CBFv values were lower in older adults at baseline.
Neutral activation					
Stefanidis [114]	TCD (MCA)	29 older, 29 younger	Older aged over 60, younger aged under 30	Ten blocks of 40 s of reading	No differences in CBFv responses between older and younger adults (22.9 vs. 21.61%).
Madureira [61]	TCD (MCA)	58 participants across 20 to 80 years	Mean age (total sample): 48	Working memory task at 1- and 2-back gain	No differences in CBFv responses at 1- or 2-back gain across age strata
Hypoactivation					
Lipecz [57]	Dynamic RVA	11 older, 18 younger	Mean ages: 75 and 33	Flicker light stimulation	51.78% reduction in mean maximal arteriolar dilation in older adults. No differences in venous dilation
Kneser [51]	Dynamic RVA	52 participants across 20–78 years	Total sample range: 20–78	Flicker light stimulation	Decrease in the arterial regulative amplitude of 45% with age and arterial constriction reduced by 75%. No changes in the venous amplitude
West [132]	BOLD-MRI	173 older, 74 younger	Mean ages: 25 and 64	Audio-visual task (checkerboard with binaural tone)	Reduced response in older adults with slower time to maximal response and return to baseline in the occipital region
Fabiani [29]	BOLD-MRI and NIRS	44 older, 19 younger	Age ranges: 65–81 and 20–28	Visual (checkerboard) stimulus	Coupling between oxy- and deoxyHb decreased with age. Haemodynamic responses were reduced in the older group. Greater variability in activation patterns of older adults (oxy-Hb).
Kannurpatti [46]	BOLD-MRI	12 older, 12 younger	Mean ages: 58 and 24	Cognitive (digit symbol verification) and motor (finger tapping) tasks	Mean activation volume reduced by 45% (motor), 40% (cognitive). Variability in activation volume was higher in older adults for the motor but not a cognitive task.
Vermeij [129]	fNIRS	14 younger, 14 older	Mean ages: 26 and 70	Working memory (verbal n-back: 0-back, control; 2-back, high working memory load)	Very low-frequency and low-frequency oscillations were reduced in older compared to younger adults during task performance. In younger adults, this increased with cognitive load.
Nowak-Flük [76]	TCD (MCA and PCA)	9 older, 10 younger	Mean ages: 66 and 23	Visual stimulation (reading)	NVC responses were reduced in older adults at rest (13.9 vs. 23.1%) and during exercise (20.6 vs. 27.4% and 16.6 vs. 24.4%) but not different between rest and exercise.
Flük [30]	TCD (MCA and PCA)	20 older, 10 younger	Mean ages: 64 and 30	Visual stimulation (checkerboard)	CBFv responses were reduced in the older group but this was not associated with arterial stiffness.
Zaletel [138]	TCD (PCA)	14 older, 26 younger	Mean ages: 70 and 38	Visual (checkboard) stimulus at 100, 10 and 1% visual contrasts, and visual evoked potentials	Younger adults had higher flow responses (139, 119 and 129% higher at 100, 10 and 1% visual contrasts). NVC index was reduced in older adults.
Orlandi [80]	TCD (MCA)	25 older, 30 younger	Mean ages: 67 and 26	Simple motor tasks	In older adults, CBFv responses were reduced with less lateralisation, delayed peak response and the return to baseline was increased.

*ACA*, anterior cerebral artery; *BOLD-MRI*, blood oxygen level-dependent magnetic resonance imaging; *MCA*, middle cerebral artery; *NIRS*, near-infrared spectroscopy; *PCA*, posterior cerebral artery; *RVA*, retinal vessel analysis; *TCD*, transcranial Doppler ultrasonography

### Hyperactivation

In a recent study by this group, TCD-measured task activation using memory and visuospatial cognitive paradigms resulted in increased CBFv responses, relative to baseline, in older compared to younger individuals [7]. This is in keeping with a previous TCD study of varying cognitive exercises [26]. Similarly, Sorond et al. found greater TCD-measured CBFv responses in older adults, in response to word-stem completion and visual search tasks compared to younger adults [112]. These findings were not task specific, but the regional activations seen in the younger group were lost in the older group for the word-stem completion task [112]. Csipo et al. demonstrated larger NIRS-measured haemodynamic responses to finger tapping in the contralateral motor and prefrontal cortices in older adults [23]. Furthermore, the deoxyHb signal was delayed and reduced (effectivity of washout), with loss of the early oxyHb signal which is thought to represent mental preparation for the activity [23]. Jamardar recently demonstrated hyperactivation at four levels of difficulty in a working memory and task-switching paradigms in older adults using fMRI [42]. Hyperactivation may be a compensatory mechanism, where increased neuronal recruitment can maintain cognitive performance in line with that of healthy younger adults [106]. This is supported by a number of cognitive theories of ageing including the hemispheric asymmetry reduction in older adults (HAROLD) [8] and compensatory-related utilisation of neural circuits hypothesis (CRUNCH) [106].

### Hypoactivation

In contrast to the findings outlined above, Kannurpatti et al. found reduced BOLD-MRI NVC responses to cognitive and motor paradigms in older adults [46]. Reduction in grey matter volume accounted for the majority of this difference, but age-related vascular and neural changes differentially affected the responses to motor and cognitive paradigms, respectively [46]. In a review of TCD-measured task activation by Stroobants and Vingerhoets, one study demonstrated lower CBFv responses to motor and cognitive paradigms, with less lateralisation [80, 115]. In three studies of visually evoked TCD-measured CBFv responses in the PCA, older adults showed lower CBFv responses to visual stimulus compared to younger adults [30, 76, 138]. Furthermore, the calculation of an index of NVC (evoked flow/evoked potential) showed a reduction in NVC responses in the older group [138]. This is in keeping with two BOLD-MRI studies, demonstrating reduced neuronal and haemodynamic responses [29, 132], and delayed time to peak response and return to baseline [132]. Using dynamic retinal vessel analysis, ageing was associated with a 45–51% reduction in retinal vessel response to light stimulation [51, 57]. This reduction may be as a result of endothelial dysfunction, which has been shown to occur with ageing, resulting in a reduced ability of vessels to increment flow appropriately when stimulated [57, 60, 117]. Finally, in a NIRS-measured study using a working memory task at two difficulty levels, oscillations at low and very low frequencies were reduced in older compared to younger adults during task performance [129]. In younger adults only, this increased with cognitive load [129].

### Neutral activation

Contrary to the studies described above which demonstrated hyper- and hypoactivation of NVC responses in ageing, a number of studies have not been able to demonstrate any discernible effects of ageing on NVC. In a study of 29 healthy older adults aged over 60 and 29 younger adults aged under 30, Stefanidis et al. did not identify age-related differences in CBFV response to ten blocks of 40 s of reading [114]. Similarly, Madureira et al. found no differences in TCD-measured NVC using a working memory task [61]. Grinband et al. found no differences in BOLD-MRI measured responses to two audio-visual tasks between older and younger adults [34]. The authors concluded that age-related changes in the haemodynamic response activation are more likely to be related to neural-cognitive rather than vascular changes [34].

### Summary of NVC changes in ageing

The inconsistent findings on the effects of ageing on NVC could be due to methodological differences, such as the type of neuroimaging technique used, the nature of the participants in the study, the paradigm used for neuro-activation and the number of times the paradigm was presented. For example, sex and handedness can have a significant effect on the lateralisation patterns of NVC responses as measured by TCD [115]. The upper cutoff for what is considered “old” varies considerably between studies [109]. Traditionally, older adults have been considered anyone over the age of 65 [109], but from a vascular perspective, changes are seen from the fourth decade onwards [25], and the age at which changes in NVC begin to occur remains unknown. Studies investigating NVC tend to be limited by small sample sizes [23] and employ case-control designs which are prone to selection bias, particularly where changes are compared at the extremes of the ageing spectrum [63, 115]. Studies may elect to use a block trial design with responses averaged over repeated trials or event-related design where single-event trials are used [20, 96]. Block designs improve signal to noise ratio, but responses can be accommodated with repeated stimulation [20, 96]. Therefore, results may vary between studies using block or single-event designs. Depending on whether studies investigate localised or broader haemodynamic responses may affect whether increases or decreases are seen in NVC responses. For example, whilst localised responses may be impaired, compensatory rises may be seen in other brain regions as a result of upregulation of existing processes, recruitment of additional neural circuits (selection) or the generation of novel neural circuits (re-organisation) [8, 16, 101]. Certainly, this has been highlighted as a key feature of the CRUNCH hypothesis of ageing, whereby neural circuits are re-organised to utilise wider brain resources to compensate for declining processing efficiency and maintain cognitive performance [8, 101].

Hyperactivation of NVC responses has been demonstrated by a number of studies and was traditionally thought to occur as a result of rising metabolic demands due to compensatory recruitment or upregulation to maintain behavioural performance [8, 27, 42, 101]. However, recent work has challenged the notion of compensatory hyperactivation, which may instead be a maladaptive process due to inefficient resource utilisation and processing [16, 70, 73]. In true compensatory hyperactivation, there should be a demonstrable correlation with improvement in cognitive performance [16]. It is important to distinguish haemodynamic task activation as a result of sympathetic stimulation (causing rises in BP and heart rate), metabolic or O_2_ feedback and that due to central command due to neuronal signalling [62]. Maggio et al. demonstrated the presence of a small rise in CBF to elbow flexion during hypercapnia despite CBF far in excess of the NVC demand as a result of the increased supply from CO_2_-induced vasodilation [62]. This finding suggests that central command through neuronal signalling may be driving the NVC response, rather than via a feedback mechanism from metabolic or O_2_ demand [62]. In support of this, a recent review identified up to 30% of the NVC response remains unaccounted for in terms of mechanistic pathways [38]. However, the effects of ageing on NVC in hyper- and hypocapnic states have not yet been investigated. Hyperactivation in response to cognitive stimulation is also seen in mild cognitive impairment (MCI) [5], where patients have an 80% chance of developing dementia over five years [95]. Thus, although hyperactivation may be present in cognitively healthy individuals, it may not represent a normal physiological adaptation to ageing. Longitudinal fMRI studies predicting cognitive decline from task activation have shown mixed results, with some demonstrating hyperactivation [134] and others demonstrating hypoactivation [10, 36, 78, 94] as protective of cognitive function. The differences in these findings are likely due to heterogeneity introduced by small sample sizes, inclusion of those with genetic risk factors (i.e. APOe4 allele), and the choice of cognitive paradigm [134]. Hypoactivation may occur in the context of vascular ageing, due to increasing arterial stiffness and reduced compliance, reducing the ability of arteries to rapidly dilate in response to stimulation [25, 29, 137, 138]. Therefore, specific patterns of response may not always be “healthy” despite intact cognitive function and can be indicative of future cognitive impairment risk. Further work is required to investigate the relationship between neuroimaging features of ageing and future risk of cognitive decline to identify protective versus maladaptive haemodynamic ageing patterns.

According to the CRUNCH hypothesis, more cognitively challenging paradigms (i.e. episodic memory) are more likely to result in hypoactivation than less challenging paradigms (i.e. semantic memory), due to a ceiling at which compensation is no longer effective [101, 134]. Thus, hypo- or hyperactivation may be seen depending on the level of difficulty introduced by the cognitive paradigm [42]. In addition, the type of cognitive paradigm could also be important, given that ageing disproportionately affects the speed of processing, working memory and executive functioning [71]. Thus, tasks involving these elements may result in hyperactivation, whereas those less affected by ageing may result in neutral or hypoactivation. In Table 1, the majority of tasks demonstrating hypoactivation used a visual stimulus, focusing on the posterior cortex, rather than more complex higher cortical functions. However, this notion was recently challenged by Jamadar, who demonstrated hyperactivation in older adults at all levels of task difficulty [42], exceeding that which was tested in the original CRUNCH model [101]. For future studies, a range of paradigms and difficulties may be required that test different aspects of cognitive function but also different functional and structural brain regions. In this regard, combining neuroimaging techniques with good spatial and temporal resolution will provide important insights into the physiological changes that occur in NVC in ageing, and whether there is region specific or time dependent [23].

## Cerebral autoregulation

### Relevance of haemodynamics and the healthy brain

CA is a measure of haemodynamic integrity of myogenic mechanisms with co-existing metabolic and neurogenic components [100, 133]. It allows the cerebral perfusion and brain tissue oxygenation to be buffered against BP changes [124]. In most studies of CA, CBF has been measured using CBFv as a surrogate [56].

CA can be expressed as a static or dynamic mechanism. Static CA regulates CBF over long-term changes in cerebral perfusion pressure (CPP) (minutes to hours) [92]. Cerebral vasculature may have the more efficient autoregulatory ability when mean arterial pressure (MAP) is increased than when decreased [77]. Static CA can be measured by manipulating blood pressure (BP) and measuring the autoregulatory response by the change in CBF. CA is intact if blood flow is maintained at or near the baseline level [121], where static CA demonstrates a nearly constant CBF for MAP changes from 60 to 150 mmHg [84]. If a change in BP leads to a significant change in CBF, CA is impaired [121, 133]. Although the static approach can evaluate the overall effect (efficiency) of CA, it does not reflect the latency of the response and it is also very difficult to implement, due to the need to use vasoactive drugs to achieve stable changes in MAP [1, 121].

Dynamic CA (dCA) is the pressure-flow relationship seen during transient changes in mean arterial BP, over a period of seconds [1]. Measuring dynamic CA involves inducing rapid changes in MAP caused by BP manoeuvres such as the sudden release of compressed thigh blood pressure cuffs (as an autoregulatory stimulus) and comparing BP and CBFV during the autoregulatory response [121]. dCA measurement gives information about the latency, as well as efficiency and can be studied with a number of different manoeuvres to induce rapid changes in MAP [121].

### Introduction to cerebral haemodynamic indices in the healthy brain

The dynamic relationship between mean BP and CBF (or CBFv) has been modelled in the time or frequency domain [22, 81, 127], giving rise to a number of different parameters or indices that reflect the efficiency and/or latency of the CA response [22, 81, 127]. Examples of dCA indices obtained in the time domain are the correlation coefficient Mx or the autoregulatory index ARI. Frequency domain representation of the BP-CBFv relationship is often obtained with transfer function analysis (TFA), which generates measures of coherence, gain and phase, often used as indices of dCA efficiency [22, 127]. Table 2 summarises the most common indices used to measure dCA; for a more complete description of dCA indices, we direct the reader to a number of excellent reviews and papers [19, 54, 59, 84, 104, 105, 110, 125–127].

**Table 2:** A summary of the most commonly measured indices for dCA

dCA Index	Measurement	Interpretation
ARI	Measures response of CBF to rapid changes in BP Second-order linear differential equation, with 3 main coefficients that are then tabulated to correspond to 10 different values of ARI [121]	Range from 0–9 Higher ARI = better autoregulation, so faster return to baseline after MAP increase Healthy controls = 5 ± 1
Mx, Mxa	Measures dCA by calculating a time-average Pearson correlation coefficient between slow fluctuations in CBFV and CPP (Mx) or mean arterial BP (Mxa), over a given time period	0 or less suggests CA intact 0.3–0.5 suggests impaired CA
PRx	Correlation between slow-wave changes in MAP and ICP	Coefficient ranging from − 1 to + 1 Positive PRx indicates passive behaviour of cerebral vessels, so reduced CA Negative PRx indicates normally reactive vascular bed, so intact CA
RAP	Index of CVR.A CVR.A = BP/CBFV It is the inverse of the regression slope of CBFV vs BP The dynamic autoregulatory response occurs through adjustments in RAP [87]	More indicative of myogenic activity and cerebrovascular resistance [88]
RoR	Measures the rate of change in CVR, which is dependent on the change in ABP RoR = (ΔCVR/Δtime)/ΔABP Full restoration of CBF where ΔCVR = ΔABP	Increasing RoR represents an increased per second adjustment of the change in CVR to fully compensate for the change in ABP [1] Higher RoR = better autoregulation
TFA gain	Ratio of amplitude of oscillations in CBFv (output) and the amplitude of oscillations in MAP (input) at each frequency	Higher gain = poorer autoregulation (i.e. reduced ability of dCA to dampen the effects of BP on CBFv)
TFA phase	Phase measures the delay of CBFv oscillations, relative to corresponding oscillations in MAP at each frequency [127].	Higher phase = better autoregulation (i.e. changes in CBFv recover faster than those in BP) [127]

*ARI*, autoregulation index; *PRx*, pressure reactivity index; *Mx*, mean flow index; *RAP*, resistance area product; *RoR*, rate of regulation; *TFA*, transfer function analysis

### Methods of assessing dCA in healthy individuals and association with ageing

dCA can be assessed in healthy individuals by a variety of methods. These include manoeuvres to induce rapid changes in MAP, such as the thigh-cuff manoeuvre, hand-grip, squat-stand or sit-to-stand protocols. Spontaneous fluctuations in MAP can also be used, normally in conjunction with TFA. Here we will give an overview of these methods with relevance to studies of ageing.

#### TFA

TFA is widely used across studies of autoregulation to allow the estimation of dCA parameters from spontaneous rather than induced BP fluctuations [127]. For research studies focusing on older participants, this is particularly advantageous as this can be better tolerated than the thigh-cuff and squat-stand manoeuvre [4]. In a longitudinal study of ten subjects who were followed for 10 years, ARI measured by TFA was found to decrease, suggesting dCA became less efficient with age [13]. As measures were repeated in the same individuals, this study was able to overcome inter-subject variability that confounds studies of dCA [13, 104, 105]. Carey et al. used multiple methods (spontaneous, thigh-cuff release, Valsalva manoeuvre) to determine the ARI in older and younger adults [17]. Across all methods, there was no effect of age on ARI [17]. In a study of three age groups (20 young (~ 24 years), 20 older (~ 66 years) and 18 older old (~ 78 years)), only phase in the very-low-frequency range was higher in the younger compared to older but not older old adults [82]. The gain was increased in the high-frequency range of the older old compared to younger adults, but there were no other differences between other age groups [82]. Similarly, in a large database study of 129 adults (median age 57), the gain was significantly associated with age (gain increased with age) but not phase or ARI parameters [91]. In contrast to these findings, Vermeij et al. did not find any significant effect of age on NIRS-measured phase and gain [129], and Teixeira et al. found no effect of age on TCD-measured phase and gain parameters [119].

#### Thigh-cuff manoeuvre

The thigh-cuff manoeuvre can be used to rapidly alter BP. Bilateral thigh cuffs are inflated to above systolic BP. Parameters are measured throughout, including MAP and TCD of the MCA. The thigh cuffs are then rapidly deflated, which induces step decreases in MAP [1]. The CBFv and BP values after the cuff release can be used to calculate ARI [1, 121]. Furthermore, change in CVR per second, in relation to the change in BP, is referred to as the rate of the regulation (RoR) and can be used as an index of CA [1, 121]. In a study of 27 subjects ≤ 40 years and 27 subjects ≥ 55 years, an association between increasing age and dCA was not seen during transient and induced BP stimuli [17]. Specific investigations of the cerebrovascular effects of the thigh-cuff manoeuvre in older adults have demonstrated a dominance of myogenic mechanisms, largely influenced by associated EtCO_2_ changes with components of the autonomic nervous system and baroreflex exerting concomitant effects [89].

#### Hand-grip manoeuvre

The hand-grip manoeuvre (HG) uses the contraction of forearm muscles to induce changes in HR, BP and CO_2_ [99]. It causes changes in CBF, possibly due to bilateral activation of cortical brain areas involved in muscle contraction and autonomic regulation [44]. However, the use of the hand-grip manoeuvre to assess dCA assumes that the handgrip itself would not disturb dCA [48]. Jorgensen et al. found that there was not an increase in cerebral perfusion caused by the increase in BP induced by handgrip [44]. In addition, ARI is not constant during the hand-grip manoeuvre, with significant dips at the beginning and the end [86]. In a study by Carey et al. described above, there were no differences in ARI as measured by HG in older compared to younger adults [17]. In keeping with this finding, Bronzwaer et al. did not demonstrate a significant effect of ageing on dCA assessed by HG or lower body negative pressure [14].

#### Squat-stand manoeuvre

Large changes in BP can be produced from squatting from the standing position [9]. Birch et al. asked volunteers to perform cycles of squatting and standing, as indicated by a computer, which then allows changes in MCA velocity and dCA to be measured. They found that oscillations in BP led to induced oscillations in the MCA velocity, which may indicate functioning autoregulation [9]. In the squat-stand manoeuvre, there are relatively large changes in BP and hence, repeated squat-stand manoeuvres can be used to investigate the directional sensitivity of CA [90]. However, in the older population, squat-stand manoeuvres may not always be practical, with concomitant osteoarthritis, reduced exercise tolerance and comorbidities [82, 128]. Thus, many studies of older adults instead use the sit-to-stand manoeuvre, which also generates a transient response of CBF to changes in BP, if measurements are taken immediately after standing up [64, 128]. Recently, an older group of individuals (aged 50–71 years) as compared to a younger group (20–34 years) demonstrated lower ARI during maximal depth squats as compared to shallower squats—thereby suggesting less efficient dCA in older individuals during maximal depth squats as compared to rest or the shallower approach [4]. Similarly, reductions in NIRS-measured frontal cortex oxygenation were seen in 27 healthy older adults during sit-to-stand [65, 66], and high-frequency dCA was impaired in older adults during sit-to-stand [74]. However, in a study of repeated sit-to-stand manoeuvres in 58 participants (20 young, 20 older and 18 older old), there was a small reduction in phase and rise in gain with increasing age, but the authors conclude that dCA remains intact with ageing [82]. Similarly, a study of 136 adults between 21 and 89 years of age using sit-to-stand manoeuvres demonstrated higher gain at rest in older adults compared to younger and middle-aged adults but no difference in gain between age groups during sit-to-stand manoeuvres [135]. These findings were in keeping with that at Sorond et al., who also found no age-related changes in dCA with sit-to-stand in either the MCA or PCA but did demonstrate a smaller vasodilatory response in the PCA territory of older adults, suggesting it may be more vulnerable to hypoperfusion [111]. In two studies of head tilt to 30 and 70 degrees, neither showed a significant effect of ageing on cerebral haemodynamics, before, during or after tilting [18, 28]. Finally, in a study by Lipsitz et al., older normotensive and hypertensive older adults both demonstrated intact dCA during a sit-to-stand manoeuvre, as measured by TFA gain and phase [58].

**Fig. 5:**
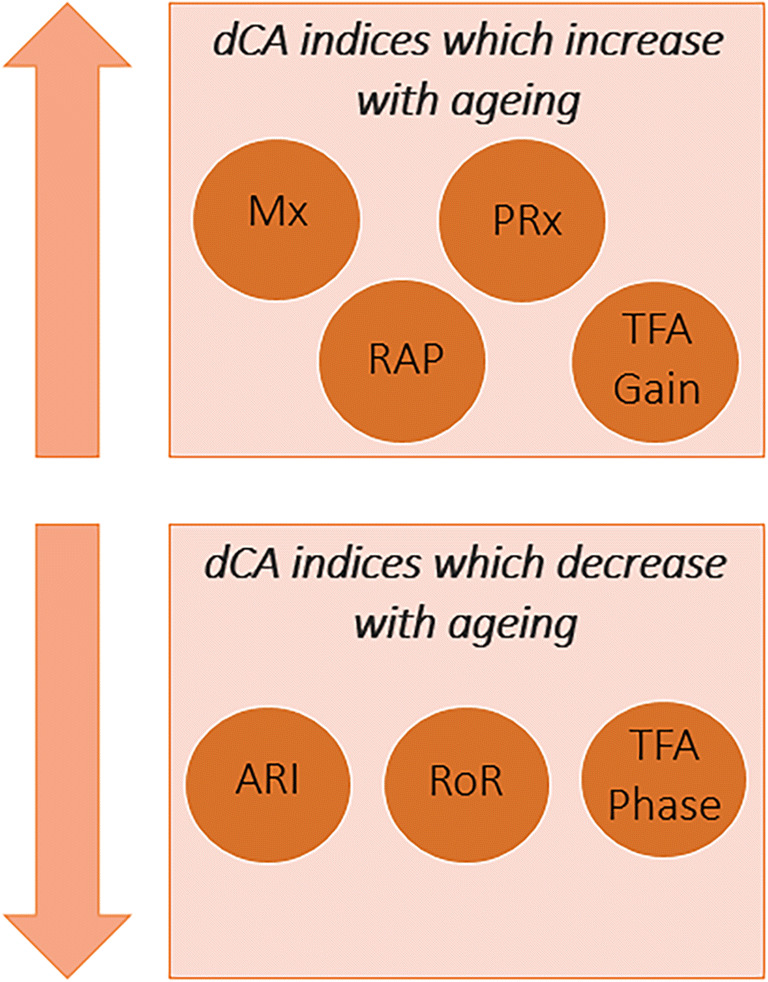
Representation of how each dCA index changes with age. ARI, autoregulation index; PRx, pressure reactivity index; Mx, mean flow index; RAP, resistance area product; RoR, rate of regulation; TFA, transfer function analysis

The various methods of assessing CA described above have provided mixed evidence of haemodynamic changes during ageing as assessed using non-respiratory paradigms. The majority of studies using sit-to-stand manoeuvres and resting TFA do not show any demonstrable effects of ageing on dCA, but there are some exceptions, specifically, the thigh-cuff manoeuvre [89] and the squat-stand measurement [4]. These conflicting findings may be due to methodological differences (resting vs induced and squat-stand vs sit-stand), and differences in the age ranges and population characteristics studied. Figure 5 summarises the changes in key dCA parameters with ageing.

### PaCO_2_, cerebral haemodynamics and brain ageing

In the context of respiratory paradigms, a large study (150 participants) recently suggested that ageing is associated with lower CBFv, greater cerebrovascular resistance and reduced vasoconstriction during hypocapnia, though increased vasodilatory responsiveness during hypercapnia [122]. Despite a longstanding acceptance that healthy ageing associates with lower CBFv [3, 127], our confidence in concluding the specific behaviours of CA indices has been more contentious. In particular, previously mentioned haemodynamic parameters are pertaining to cerebrovascular tone (critical closing pressure—CrCP), cerebrovascular resistance (RAP) [68, 69] and the ARI. Firstly, assessment of normative values and the influence of age within a large haemodynamic database demonstrated a lack of an association between increasing age and ARI during normocapnia [17, 91]. Secondly, during normocapnia, older normotensive individuals have greater RAP than younger individuals, though, during posture change, there is a greater relative change in CrCP during upright posture in younger adults [83, 102]. There is some suggestion of heightened sensitivity to postural changes through CrCP in younger compared to older individuals [102]. Thirdly, tangible changes in CVR are thought to occur in those over 50 years with the very old (> 80 years) seemingly exhibiting similar responses to those over 50 years of age; this is an important consideration when determining vascular neurological risk assessment [47].

Despite the contention generated through studies on the influence of ageing during normocapnic conditions, greater clarity has been gained through aligned hypocapnic studies of the influence of PaCO_2_ changes and healthy ageing.

#### Carbon dioxide and cerebral blood flow

Carbon dioxide (CO_2_) has a profound effect on mediating CBF. The relationship between the partial pressure of carbon dioxide in arterial blood (PaCO_2_) and CBF has been described as a sigmoid curve with lower and upper plateaus [116]. Elevated PaCO_2_ causes vasodilatation, increasing CBF, and reduced PaCO_2_ causes vasoconstriction, decreasing CBF. This is thought to be due to increased CO_2_ leading to increased [H+] resulting in vascular smooth muscle relaxation [21]. This cerebral vasomotor reactivity (CVMR) is an important mechanism that regulates brain pH levels and affects respiratory central chemoreceptors. Additionally, there are thought to be other agents involved such as prostaglandins and nitric oxide [12, 93].

#### CVMR and ageing

As discussed, ageing is associated with lower CBF and CBFv [3, 139]. However, research has yielded conflicting results regarding ageing and CVRM (i.e. response of CBF to changes to PaCO_2_) [31, 41, 136].

Zhu and colleagues found that older participants had lower CBF velocities and higher cerebrovascular resistance index in resting conditions [139]. Older participants had a reduced vasoconstrictor response to hypocapnia but increased vasodilatory response to hypercapnia [139]. This may suggest that ageing is associated with increased cerebral vasoconstrictor tone at rest, thus reducing the capacity of vessels to constrict in response to hypocapnia and increasing the capacity of vessels to dilate in response to hypercapnia. However, research remains inconsistent, with further studies finding no significant difference in CVRM or decreased response to hypercapnia [30, 72, 120]. In contrast to Zhu et al. [139], Galvin and colleagues found increased CVR to hypocapnia, correlated with increasing age, suggesting that increased CVR to hypocapnia may be contributory to the increased risk to cerebral ischaemia in ageing [31]. The varying response to CO_2_ may be related to the differences in study protocols employed in these studies, with variations in stimulus, protocols for CVR assessment and analysis. However, convergent findings do exist, though the key influencing parameter is debated. Minhas et al. (2019) did not demonstrate an alteration in ARI with CO_2_ change and increasing age, confirming prior large database findings [68]. However, an elevation in RAP, and not CrCP, was seen during a hypocapnic stimulus in older (> 50 years) as compared to younger individuals (≤ 49 years) [68]. These findings align with Ogoh and colleagues who showed in older normotensive adults that RAP is elevated but not CrCP [79]. Specifically, the data suggest that RAP and CrCP maintain CVMR during hypocapnic challenge during healthy ageing.

## Summary

Taken together, the majority of studies do not show a significant effect of ageing on dCA, despite differences in techniques and methodologies, populations and outcome measures. This poses a number of important questions. Particularly for the mechanisms of orthostatic hypotension, given that dCA remains intact during postural changes in many studies, but remains a significant contributor to falls and morbidity in the older populations. Importantly, the challenge provided by the unique influence of CO_2_ on the cerebral vasculature has arguably provided the greatest information, allowing us to differentiate the individual effects of CVMR, tone and autoregulation. Several studies have found a varying CVMR response to CO_2_ with both reduced and increased CVMR to hypercapnia and hypocapnia noted. Further research is required to elucidate the relationship between CO_2_ and CBF in the healthy brain and ageing. Developing a further understanding of the effects of ageing on the CBF dependence on PaCO_2_ in the healthy brain may help to differentiate age-related variations from abnormal changes and thus help to identify those at higher risk of cerebrovascular disease.

Studies of ageing-related NVC changes have found mixed results of hyper-, hypo- or neutral activation. These differences are likely to be due to significant heterogeneity in neuroimaging techniques and methods, paradigm selection, presence of vascular ageing and genetic risks, and small sample sizes. Aligning methodologies and techniques through the establishment of guidelines for the conduct of studies investigating NVC will facilitate comparisons and meta-analyses of findings. Where possible, studies should use multiple imaging techniques to quantify flow measurements and provide information on the spatial and temporal nature of the response. Despite these limitations, alterations in NVC with ageing have been demonstrated and correlate with cognitive performance and can predict future dementia risk. Thus, understanding “healthy” ageing patterns in cerebral haemodynamics is imperative to promoting and maintaining brain health in later life.
